# Supplementary figures and images for: Quorum Quenching Enzyme (PF-1240) Capable to Degrade AHLs as a Candidate for Inhibiting Quorum Sensing in Food Spoilage Bacterium Hafnia alvei
Source: Foods. 2021 Nov 5;10(11):2700. doi: 10.3390/foods10112700 (PMC8622684; doi:10.3390/foods10112700)

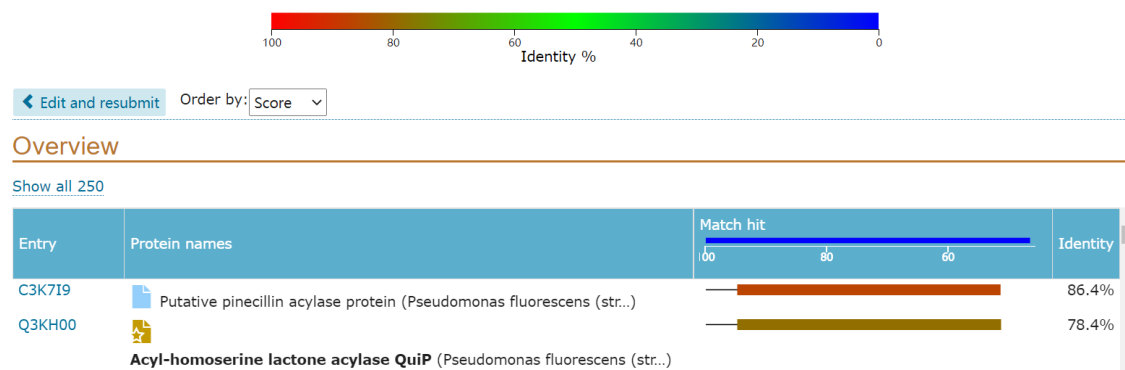

Figure S1. PF-1240 sequence alignment.

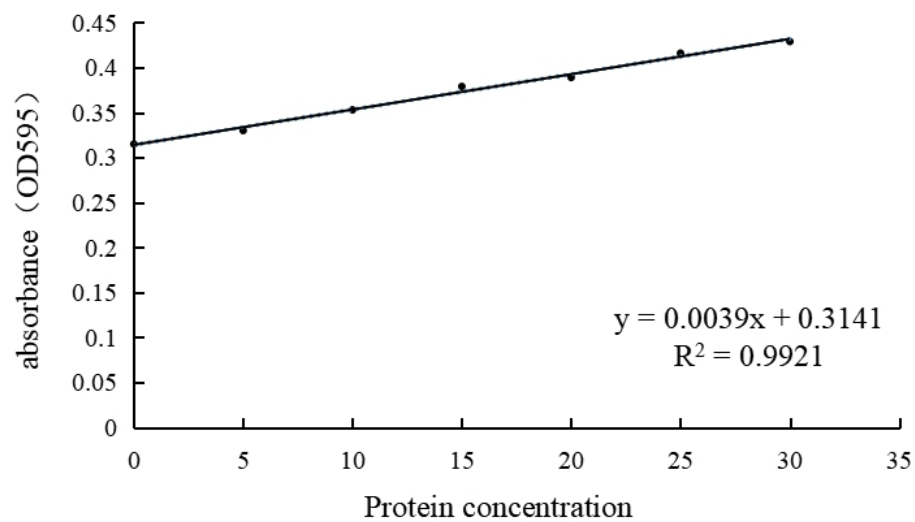

Figure S2. Protein concentration standard curve.

Supplement: Supplementary file 1 [file foods-10-02700-s001.zip › foods-1386557-supplementary.pdf]
